# Mortality, structure, propagation, and microhabitat characterization of *Haageocereus acranthus*: a case study on coastal lomas

**DOI:** 10.3389/fpls.2025.1577533

**Published:** 2025-07-30

**Authors:** Vladimir Camel, Freddy Pillpa, Virginia Colqui, Jose Ataucusi, July Quispe-Huañahue, Edwin Felix, Zulema Ninanya-Parra, Key Maravi-Hinostroza, Keiko Caysahuana, Rita Cabello-Torres

**Affiliations:** Grupo de Investigación en Ecofisiología Vegetal y Restauración de Ecosistemas Degradados, Escuela de Ingeniería Ambiental, Universidad Cesar Vallejo, Lima, Peru

**Keywords:** arid ecosystems, Cactaceae, cytometry, degraded soils, ecological restoration

## Abstract

The *Haageocereus* genus includes endemic species found in the coastal region of Peru and is characterized by varying ploidy levels that influence its shape and adaptability. It establishes itself in coastal lomas and desert ecosystems, capturing moisture from fog and reproducing through stem fragmentation and seeds to survive. Ecologically, it helps stabilize the soil and provides shelter and food for wildlife. The study aimed to propagate, evaluate mortality and structure, and characterize the microhabitat of *Haageocereus acranthus* in the coastal lomas of Mangomarca, Lima, Peru. To achieve this, three transects were established across an altitudinal gradient. The abundance, morphological data, and environmental factors (pH, cover, slope, organic matter, etc.) were assessed, and living and dead colonies were counted. Consequently, the stems grow approximately 4.8 cm per year under nursery conditions, while the roots develop 4.42 cm in 45 days. In a 4.41 ha area, 94 colonies were recorded, comprising 1,801 stems; 37.89% of the individuals had lengths between 20 and 40 cm. The largest stem reached a diameter of 8 cm and a length of 169 cm. Additionally, around 1,788 living colonies and 14,741 dead colonies were counted across all the lomas. The death of the cacti may be linked to anthropogenic pressure that has altered the soil from acidic to basic, increasing electrical conductivity while reducing the availability of organic matter and nutrients. Our research has also shown that pH and altitude influence the phenotypic characteristics of *H. acranthus* stems. At higher elevations, the size of the cacti increased alongside the amount of organic matter, while the concentration of carbonates decreased. Ultimately, mortality rates will likely rise due to severe human impacts, increasing temperatures, and prolonged droughts. Therefore, it is crucial to closely monitor and implement conservation and restoration measures for these coastal lomas endemic to South America.

## 1 Introduction

Cacti have their center of origin and most abundant diversity in the American continent, and they are used as a model to understand the origin of arid biomes. Suggesting that the most extensive cactus lineages were established in the context of climate change and the expansion of arid and semi-arid habitats (Arakaki et al., 2011). Cacti are dicotyledonous, xerophytic plants that are resistant to extreme climatic conditions. Their roots are shallow, and their metabolism is CAM-type. They live in saline or slightly acidic soils (Lim et al., 2019). There are 262 species in 40 genera in Peru (Ostolaza, 2014). Most of these taxa are distributed between 0 and 4,500 meters above sea level and mainly inhabit desert shrublands, grasslands, lomas, and coastal deserts (Arakaki et al., 2006). Currently, little is known about the conservation status of Cactaceae, which is why it is important to develop ecological studies on their structure, functioning, and restoration (Winchell et al., 2022). Various cactus species are experiencing changes in their populations. Even though they are growing, there remains a risk of increased mortality if natural regeneration rates are impacted by climatic variations and human pressures such as fragmentation, roads, particulate matter, mining activity, and urban expansion (Flores and Van Meerbeek, 2024). Therefore, given the importance of the ecosystem services they provide and their evident degradation, the regional government of Lima, Peru (2012-2025) has established as a primary objective the conservation, protection, and restoration of the urban ecosystems that constitute the ecological structure of Lima, affirming the sustainable use and recovery of degraded environments populated by abundant herbaceous, xerophytic, and stationary vegetation (Flood Chávez and Niewiadomski, 2022).

In this research, we address the genus *Haageocereus*, which includes 20 accepted species, of which seven are endangered. They also have different ploidy levels. *Haageocereus tenuis* F. Ritter (2 N = 3 x = 33) and *H. repens* Rauh and Backeb. (2 N = 2 x = 22) have a single population near the city of Lima. While *H. acranthus* (Vaup.) Backeb. (2 N = 4 x = 44) and *H. pseudomelanostele* (Werdermann and Backeb.) Backeb. (2 N = 2 x = 22) have several large populations (Arakaki et al., 2007). Its biogeographic distribution is restricted to the Pacific basin of the Andes. They are species that adapt to extremely harsh living conditions, tolerating thermal, water (annual rainfall of 18–100 mm), and radiation stress (Calderón et al., 2007).

In general, cacti efficiently absorb large amounts of carbon dioxide quickly, showing great potential as carbon sinks (Torre, 2017). Ecologically, their spines capture humidity from the air and transfer it by osmosis to their tissues. Physiologically, their stomata close during the day and open at night to prevent water loss (Calhoun, 2012), thus optimizing their photosynthetic processes. Furthermore, cacti can be utilized in the food industry, as well as for vegetable fiber, cosmetics, and more (Ostolaza, 2014). In this context (Simpalo et al., 2020), reported that the vitamin C content (66.73 mg/100 g) of the fruit of *H. pseudomelanostele* is higher than that of other fruits with similar structure, such as aguaymanto, sanqui, and tuna.

Regarding ecology, studies report that for the species *Haageocereus pseudomelanostele*, *Melocactus peruvianus*, *Mila nealeana*, and *Neoraymondia arequipensis*, rocks acted as nurses in coastal ecosystems by providing a temperature lower than 1.2°C compared to bare soil, extending the soil moisture period, accumulating more organic matter, and protecting against intense solar radiation (Pisco, 2016). This created a favorable microclimate for the seeds of these species to germinate and establish themselves or for seedlings to survive the extreme conditions of the arid ecosystem (Castro-Cepero et al., 2006). Likewise, microorganisms in the rhizosphere promote the growth of cacti (Villanueva, 2018); they are involved in seed germination and flowering processes (Chávez et al., 2016); and they assist in tolerating water and salt stress (Sánchez et al., 2023). On the other hand, recent studies in coastal lomas indicate that the percentages of organic matter, nitrogen, phosphorus, and potassium in the soil are abruptly altered by human activities, which convert acidic soils into basic ones, raising the levels of electrical conductivity due to an increase in Mg and Ca (Camel et al., 2024). This phenomenon could be linked to the death of cacti and other species typical of coastal lomas.

On the other hand, regarding the reproduction of some triploid species (*H. tenuis*) within the genus *Haageocereus*, it is essential to note that they mainly reproduce through stem fragmentation and agamospermy, producing viable seeds without sexual fertilization (Arakaki et al., 2013). This indicates that all individuals are genetically identical, suggesting that the population represents a single clone. This process is crucial for plants that colonize specific areas or face adverse conditions, as it ensures genetic stability. Conversely, *H. repens* is endangered and limited to a single population (Arakaki et al., 2007). In contrast, the widely distributed species *H. pseudomelanostele* (diploid) and *H. acranthus* (tetraploid) exhibit high genetic diversity, indicating potential gene flow influenced by insects, bats, and hummingbirds (Arakaki, 2008). Additionally, the seed propagation of *H. pseudomelanostele* shows a high germination rate and healthy development under shaded conditions (Castro-Cepero et al., 2006).

Therefore, it is essential to propose restoration models for coastal lomas since they are fragile ecosystems (Balaguer et al., 2011). In this way, desertification processes can be slowed down, in addition to avoiding the extinction of endemic species under some threat. To do this, it is crucial to know the behavior of native species, such as their propagation and climatic and edaphic conditions of their microsite, and evaluate the development, physiology, and plant-microorganism interaction (Cordero et al., 2017). For this reason, the present work aims to propagate, evaluate mortality and structure, and characterize the microhabitat of *Haageocereus acranthus* in the coastal lomas of Mangomarca, Lima-Peru.

## 2 Materials and methods

### 2.1 Study area

This study was conducted in the lomas of Mangomarca, situated in the district of San Juan de Lurigancho-Lima, Peru, and encompasses an area of 516.10 hectares. It is regarded as a fragile ecosystem threatened by human activities since pre-Inca times (Marcone et al., 2024), The Ichma culture, which flourished near the lomas of Mangomarca, along with the archaeological findings, indicates that they utilized resources such as granite rocks (composed of ferromagnesian minerals, calcium-sodium feldspars, alkali feldspars, and quartz), wood, and fruits (Eeckhout, 2004). The lomas of Mangomarca are characterized by ravines and slopes exceeding 30 degrees. They are found between 180 and 850 meters above sea level and are noted for their rocky outcrops (Camel et al., 2024). Furthermore, in the coastal lomas, the existence of animals (lizards, birds, bats, and rodents) is intricately linked to perennial plants (*Solanum peruvianum*, *Trixis cacalioides*, *Atriplex rotundifolia*, *Haagacereus* sp.), which play a crucial role in their diet. These animals feed on the stems, fruits, leaves, and juicy fleshy roots. In return, they are valuable for pollinating flowers and propagating seeds (Bugaret, 2010).

### 2.2 Inventory and population structure of *Haageocereus acranthus*

**Figure 1:**
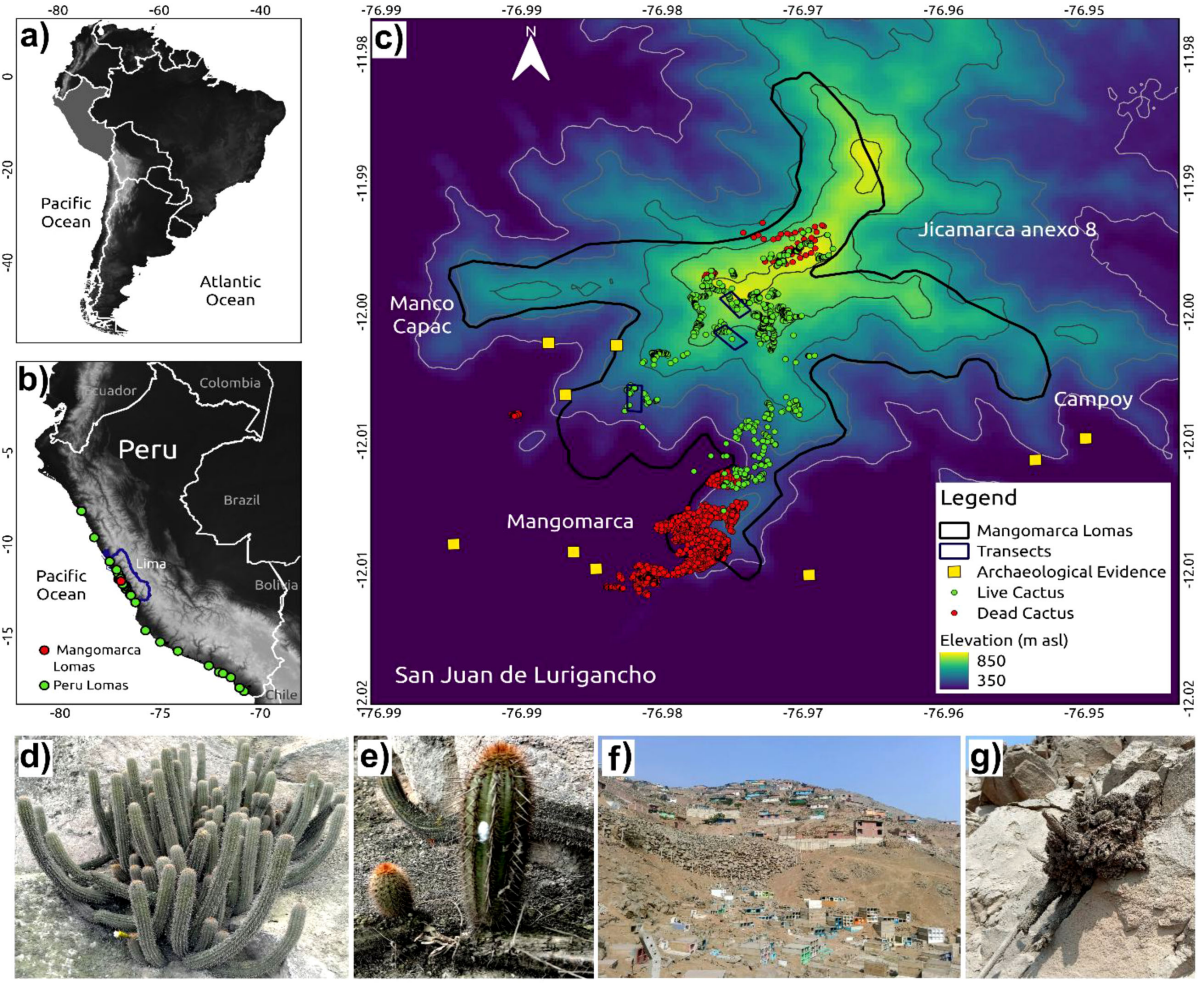
Location of coastal lomas ecosystems in Peru and spatial distribution of *H*. *acranthus* colonies. **(a)** Geographic location of Peru in the South American continent. **(b)** Distribution of lomas in Peru: the green dots indicate the location of other coastal lomas, and the red dots indicate the position of the Mangomarca Lomas. **(c)** Distribution of *H*. *acranthus* in Mangomarca Lomas: the red dots represent dead cacti, and the green dots indicate living cacti. **(d)** A colony of the Cactus *H*. *acranthus*, featuring 77 stems, is located within the preserved area of the Mangomarca Coastal Lomas. **(e)** Asexual reproduction of *H*. *acranthus*: the stem detaches from the parent plant and continues the natural rooting process. **(f)** A coastal lomas area that is being degraded by human activities. The primary activities include housing and road construction, illegal granite mining, planting exotic plants, increased animals (such as dogs) that trample the biological crust, and rising amounts of solid waste. **(g)** Dead *H*. *acranthus*, with visible signs of deterioration and decomposition.

To conduct the inventory of the *H. acranthus* cactus, three transects were established throughout the study area to analyze the structural and environmental characteristics (**Figure 1c**). Each transect was 100 m wide, with a line perpendicular to the slope established along its length. Additionally, the transects were set up at different elevation levels: T1 (0.90 ha, from 448 to 523 m asl), T2 (1.79 ha, from 674 to 769 m asl), and T3 (1.72 ha, from 760 to 841 m asl) with a slope greater than 30% (**Figure 1c**). The layout of the transects aims to inventory the cacti about altitude and slope, their variation due to the presence of rocks, and areas devoid of vegetation. Data collection was conducted *in situ*, with location coordinates recorded at a spatial level alongside slope and elevation. Furthermore, the height and diameter of the cactus stems by colony were measured. To evaluate the soil cover around each colony, the following variables were recorded: distance to rock cover, soil depth, presence or absence of trails, presence or absence of lichen, and presence or absence of solid waste. Likewise, soil samples were collected from each sampled cactus live colony. On the other hand, 50 soil samples were collected from dead cacti along the roadside, and their geographic coordinates (UTM) were recorded. And later, in the biotechnology laboratory of Cesar Vallejo University, the pH value, humidity, percentage of organic matter, and percentage of carbonates were determined.

On the other hand, to spatially locate all the living and dead cactus colonies in the lomas of Mangomarca, Aster satellite images and the GPS Garmin 76SX were utilized. All maps were processed using the QGIS program.

### 2.3 Rooting, growth, and genome size estimation

First, individuals were collected near the roads and plants that were found under anthropic pressure due to the presence of particulate matter. Subsequently, the cacti were washed to remove dust residues; the wounds were cauterized at room temperature in the shade. After 30 days, the rooting process was carried out on bare soil using the Premix5 substrate, which is composed of Canadian Sphagnum peat moss (75 - 85% approx.), perlite (20% approx.), dolomite, gypsum, silicon dioxide (0.25%), soluble silicon (0.12%), with a pH between 5.5 and 6.0, and electrical conductivity between 0.75-1.0 dS m^-1^. After 45 days, the plants were carefully extracted and transferred to pots with substrates of black soil, blond peat, and stones in a 3:2:1 ratio. On the other hand, growth measurements were made for 1 year. Flow cytometry analysis was conducted in the cereals and native grains program laboratory at the National Agrarian University La Molina. Three samples were analyzed following the protocols described by Doležel et al. (2007) and Salcedo (2022). In a cold Petri dish, 1 ml of OTTO I (100 mM citric acid, 0.50% (v/v) +/Tween 20; pH 2-3), 1 g of fresh tissue from the apical part of the *H. acranthus* stem, and 1 cm² of fresh *Physalis peruviana* L. tissue was placed as a reference standard. The entire sample was then mechanically homogenized with a double-edged blade. Next, it was filtered using a 40 µm nylon mesh and centrifuged at 1500 rpm at 5°C for 5 minutes. The supernatant was discarded, and the pellets were resuspended in 500 µl of OTTO I, followed by the addition of 500 µl of OTTO II (400 mM Na_2_PO_4_·12H_2_O, pH 8–9) supplemented with 50 µg/ml of propidium iodide (Sigma-Aldrich Company, USA) and 50 µg/ml of RNAse (Sigma-Aldrich Company, USA). The resuspension was incubated at 5°C for 10 minutes and analyzed using an Attune Nxt flow cytometer (Thermo Fisher Scientific) (Salcedo, 2022).

### 2.4 Data analysis

A linear model was used to evaluate the correlation between chromosome number and genome size. To do this, the genome size of *H. acranthus* in the Lomas de Mangomarca was estimated and complemented by other scientific reports identified through text mining. On the other hand, the correlation of root growth with the diameter and height of the stems was analyzed using generalized linear models. Next, we utilized mixed effects models to compare MO, EC, and pH in Lomas Coastal soils (disturbed and undisturbed). To account for the possible lack of independence between soil samples, we included the state of soil (perturbed and non-perturbed) as a random factor (Bolker et al., 2009). A Gaussian error distribution with an identity link function was used as the model due to the normality tested by the Shapiro–Wilk test. A Kruskal–Wallis with Bonferroni–Dunn *post hoc* test (p < 0.05) was used to compare differences (Matter organic, electric conductivity, and pH) between samples for perturbed and non-perturbed. We used generalized linear models to examine the correlation between the closest road distance to live (conserved area) and dead (disturbed area) cacti, with their respective pH values. To understand the structure of *H. acranthus*, a stacked bar histogram was made where the different colors represented the transects. The total height (cm) was considered a class because it is the most differentiated morphological trait among the other stems in the colonies. Likewise, I would like to analyze the relationship between structure, environmental variables, and anthropogenic effects. A multi-model inference approach was utilized to achieve this. Structural components, such as the number of stems per colony, diameter, and total height, were treated as response variables. Conversely, environmental conditions (including altitude, distance to the rocks, slope, humidity, soil pH, and organic matter) and anthropogenic impacts (the presence or absence of trails and solid waste) were considered explanatory variables. Transects were considered a random effect. Based on Akaike’s information criterion (AIC), we ranked the models from the best to the worst and considered the set of models with ΔAICc < 2 as equally well supported (Burnham et al., 2010). Since the response variable was a ratio, we applied a log-normal distribution.

Finally, generalized linear models were employed to assess the relationship between elevation (m asl) and variables such as stem diameter (cm), total stem height (cm), organic matter (%), carbonates (%), relative soil humidity (%), and pH. All the analyses were conducted using the R-Project software (R Core team, 2024); for the GLMM, we employed the lme4 package (effects and lmerTest) (Bates et al., 2015; Kuznetsova et al., 2017), MuMIn package for multi-model inference (Bartoń, 2010), and ggplot2 package for plots in general (Wickham, 2013).

## 3 Results

### 3.1 Determining the genome size of *H. acranthus*

**Figure 2:**
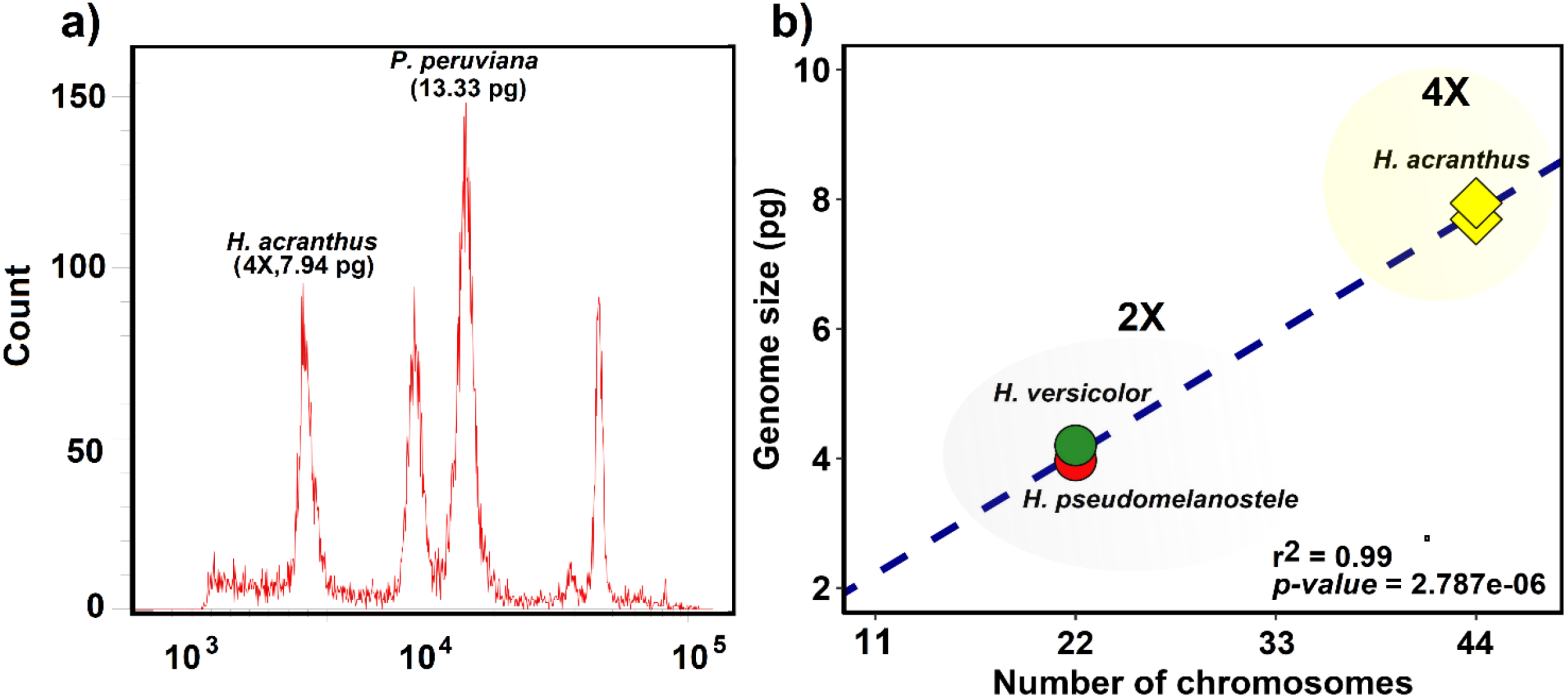
Estimating the absolute amount of nuclear DNA (genome size) in *H*. *acranthus* individuals. **(a)** Comparison of the G1 peak between *Physalis peruviana* and the tetraploid genotype of *H*. *acranthus*. **(b)** Correlation between chromosome number and genome size in three species of the genus *Haageocereus*.

The chromosome number and genome size of three *Haageocereus* species (*H. versicolor*, *H. pseudomelanostele*, and *H. acranthus*) have been recorded (**Table 1**). **Figure 2a** indicates that the genome size of *H. acranthus* from lomas of Mangomarca reached 7.94 ± 0.01 pg of DNA, confirming the correct genome size estimation for 4X tetraploid individuals with 44 chromosome pairs (**Figure 2b**).

**Table 1:** Chromosome numbers, genome sizes, and seed measurements of the genus *Haageocereus*.

Species	Ploidy	CN	GS (pg)	Seed size (mm)	Seed weight (mg)	References
*Haageocereus acranthus (Vaupel) Backeb.*	Tetraploide	44	7.94	1.51	0.53	(Arakaki, 2008; Results by V.C.)
*Haageocereus acranthus (Vaupel) Backeb.*	Tetraploide	44	7.69	1.41	0.51	(Arakaki, 2008; Silva, 2015)
*Haageocereus pseudomelanostele (Werderm. & Backeb.) Backeb.*	Diploide	22	3.97	1.28	0.33	(Arakaki, 2008; Silva, 2015)
*Haageocereus versicolor (Werderm. & Backeb.) Backeb.*	Diploide	22	4.2	1.21	0.37	(Arakaki, 2008; Silva, 2015)
*Haageocereus australis Backeb.*	Diploide	22				(Arakaki, 2008)
*Haageocereus chalaensis F. Ritter*	Tetraploide	44				(Arakaki, 2008)
*Haageocereus decumbens (Vaupel) Backeb.*	Diploide	22				(Arakaki, 2008)
*Haageocereus fulvus* var. *yautanensis*	Tetraploide	44				(Arakaki, 2008)
*Haageocereus horrens Rauh & Backeb.*	Diploide	22				(Arakaki, 2008)
*Haageocereus icosagonoides Rauh & Backeb.*	Diploide	22				(Arakaki, 2008)
*Haageocereus multangularis (Willd.) F. Ritte*	Diploide	22				(Arakaki, 2008)
*Haageocereus multicolorispinus Buining*	Tetraploide	44				(Arakaki, 2008)
*Haageocereus pacalaensis subsp. repens (Rauh & Backeb.) Ostolaza*	Diploide	22		1.2		(Arakaki, 2008)
*Haageocereus platinospinus (Werderm. & Backeb.) Backeb.*	Diploide	22			0.2	(Arakaki, 2008; Jara-Peña, 2024)
*Haageocereus pseudoversicolor Rauh & Backeb.*	Diploide	22				(Arakaki, 2008)
*Haageocereus tenuis F. Ritter*	Triploide	33		1.415		(Arakaki, 2008; Alcalá, 2021)

### 3.2 Rooting and longitudinal growth of *H. acranthus*

**Figure 3:**
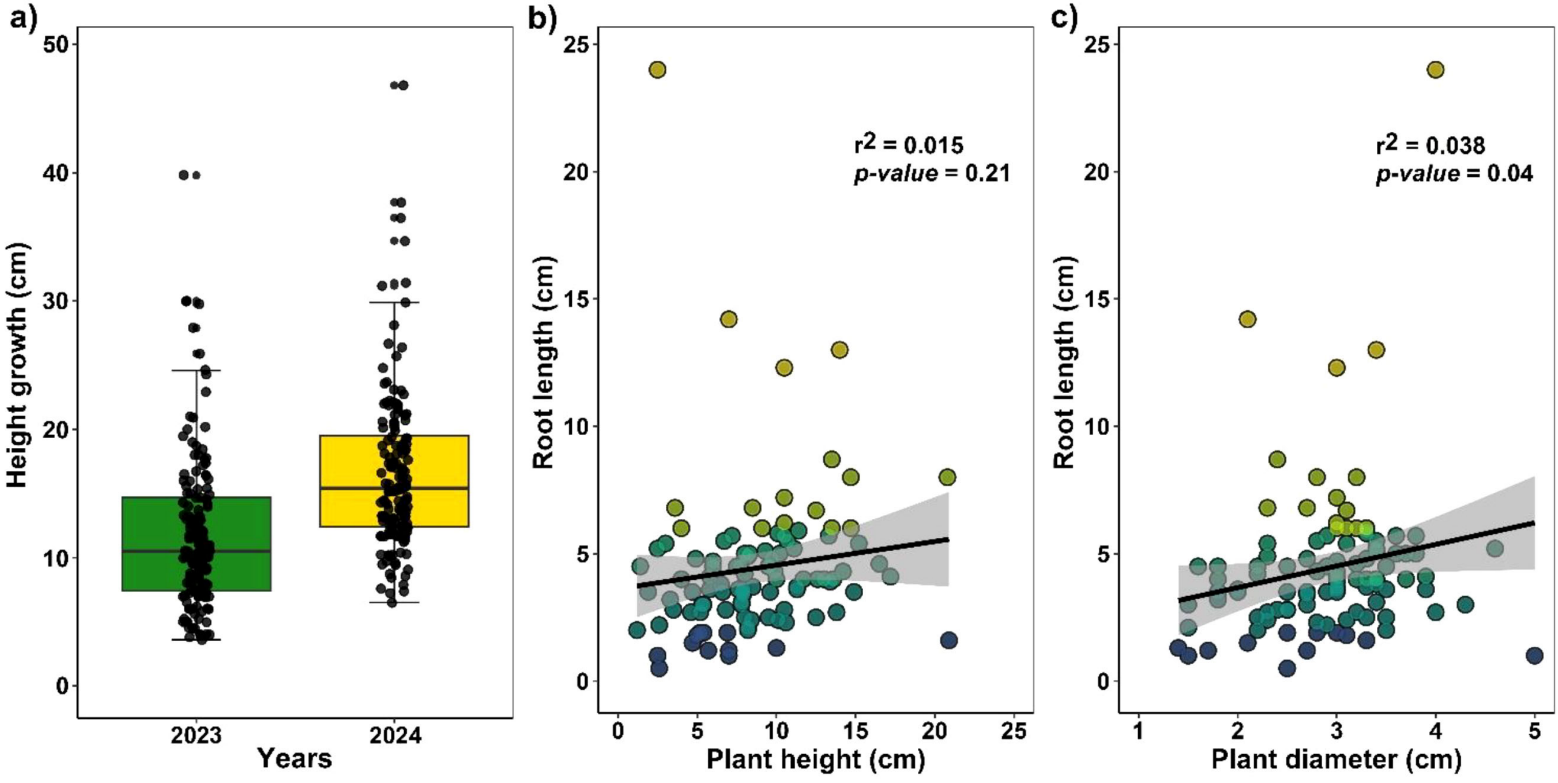
**(a)** Annual growth of cactus stem length. **(b)** Correlation between *H*. *acranthus* stem size and root length. **(c)** Correlation of *H*. *acranthus* stem diameter with root length. The rooting process occurred in bare soil for 45 days.

Under nursery conditions, *H. acranthus* stems grew an average of 4.80 ± 0.2 cm over one year (**Figure 3a**). Likewise, during the rooting process, *H. acranthus* individuals developed primary roots measuring 4.42 cm in 45 days. However, correlation analyses show that the diameter and height of the stems do not significantly affect root growth (**Figures 3b, c**).

### 3.3 Inventory and population structure of *H. acranthus*

Approximately 1,788 living cactus colonies and 14,741 dead colonies of *Haageocereus acranthus* were identified, spread over an area of 270.10 hectares within an altitudinal range of 228 to 852 meters above sea level (m asl). The highest concentration of living *H. acranthus* was observed at altitudes ranging from 500 to 800 m asl (**Figure 1**), while the dead individuals were found between 250 and 500 m asl near the human population. Additionally, **Figure 1** shows a limited presence of *H. acranthus* individuals at the northern and eastern ends of the Mangomarca hills. The results also indicate that the species primarily reproduces from stems and shows no evidence of plant propagation from seeds.

**Figure 4:**
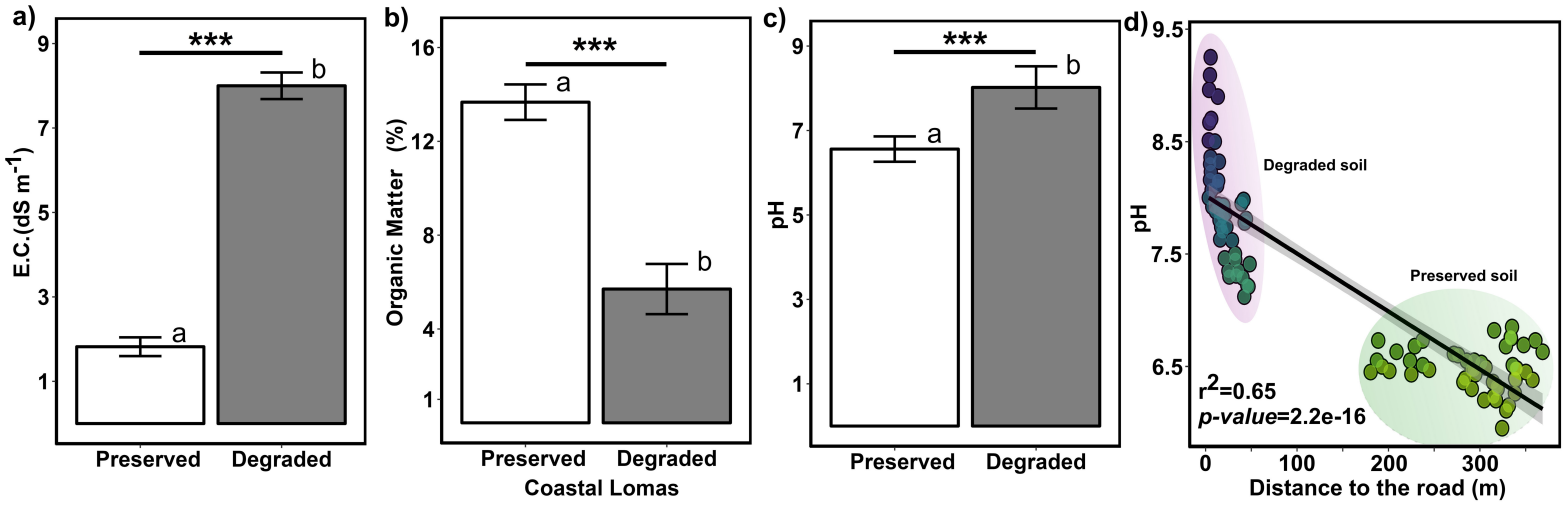
Comparison of soil properties between conserved and degraded soils. **(a)** electrical conductivity. **(b)** pH levels. **(c)** organic matter content. **(d)** Correlation between the distance of living and deceased individuals of H. acranthus to the nearest road and soil pH. The asterisks indicate significant differences between preserved and degraded soil: “*” p ≤ 0.05; “**” p ≤ 0.01; and “***” p ≤ 0.001. The letters (a, b) indicate that treatments differ significantly from each other (p < 0.05). Therefore, degradation significantly affects these soil properties (pH, E.C., and O.M.) in the Coastal Lomas.

On the other hand, the differences between the soils (electrical conductivity, pH, and organic matter) of living and dead cacti were analyzed (**Figure 4**). The results indicate that human impact significantly affected all three parameters. Regarding EC and pH, there was an increase from 1.5 to 8 dS m⁻¹ and from 6.54 to 7.68, respectively, while the percentage of organic matter decreased from 14% to 5% (**Figure 4**). We also demonstrated that the soil pH level in coastal lomas changes from acidic to alkaline as it approaches trails and roads (**Figure 4d**).

**Figure 5:**
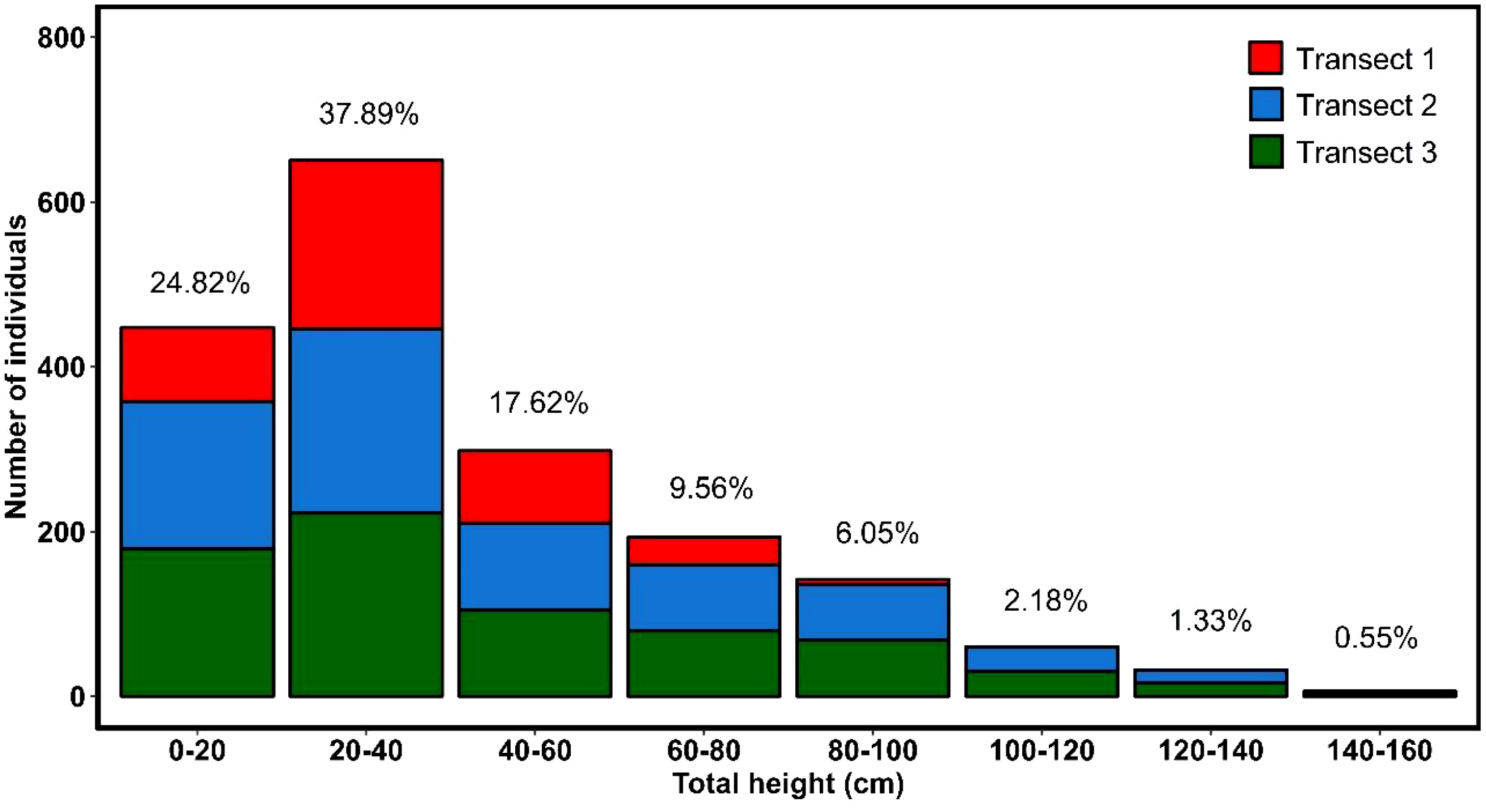
Distribution of *Haageocereus acranthus* individuals by height category across the three study transects.

The inventory of colonies and stem numbers across three transects (**Figure 5**) shows that transect 1 (0.9 ha) contains 33 colonies with 429 stems, and transect 2 (1.79 ha) has 30 colonies with 788 stems. Transect 3 (1.72 ha) records 31 colonies with 584 stems. The frequency of total height classes indicates that 37.89% of the plants fall between 20 and 40 cm, followed by those smaller than 20 cm at 24.82% (**Figure 5**). Furthermore, individuals exceeding one meter in height were observed in the higher elevation transects T2 (between 674 and 769 m asl) and T3 (between 760 and 841 m asl).

### 3.4 The influence of environmental factors on the development of the species *Haageocereus acranthus*

**Figure 6:**
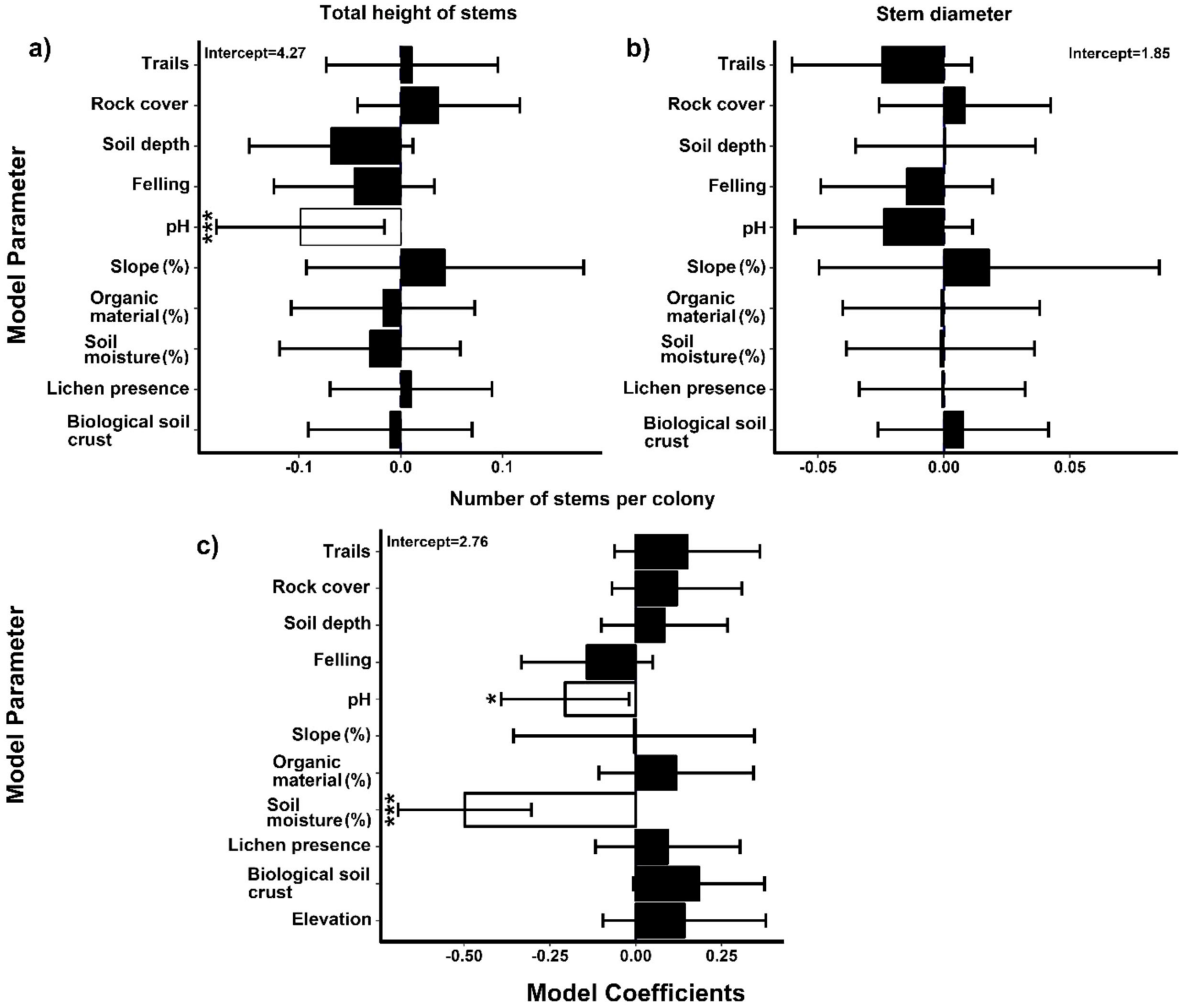
A generalized linear mixed model assesses the effects of environmental factors on the number of *H*. *acranthus* individuals in the study area concerning soil depth (SD), biological crust (BC), pruning, slope, organic matter (OM), humidity, presence of lichen, and altitude. **(a)** Total height of stems. **(b)** Stem diameter. **(c)** Number of stems in the colony. The error bars represent 95% confidence intervals. The white boxes with asterisks indicate significant effects on the structural parameters (p < 0.05): “*” p ≤ 0.05; “**” p ≤ 0.01; and “***” p ≤ 0.001.

The lomas of Mangomarca have an average slope of 46.6%. Its soil properties include a depth of 6.8 cm (between soil and organic matter) and an average pH of 6.36, with humidity reaching 10.37% in May. In terms of organic matter, it contains 12.36% (**Supplementary Material Table 1**). The results regarding the relationship between environmental and anthropogenic factors and the height, diameter, and number of stems per colony (**Figure 6**) indicate that pH significantly influences the structure of *H. acranthus* in the lomas of Mangomarca. **Figure 6a** shows that an increase in pH negatively impacts the total height of the evaluated cactus stems. Additionally, the findings suggest that as pH (*p-value* = 0.0380) and relative soil humidity (*p-value* = 5e-07) increase, the number of *H. acranthus* stems per colony decreases (**Figure 6c**). Conversely, stem diameter remained unaffected by any evaluated parameter (**Figure 6b**). Furthermore, factors such as % OM, slope, distance to rock, presence of lichen, and biological crust did not show significant relationships with the abundance of stems and phenotypic traits (height and diameter).

**Figure 7:**
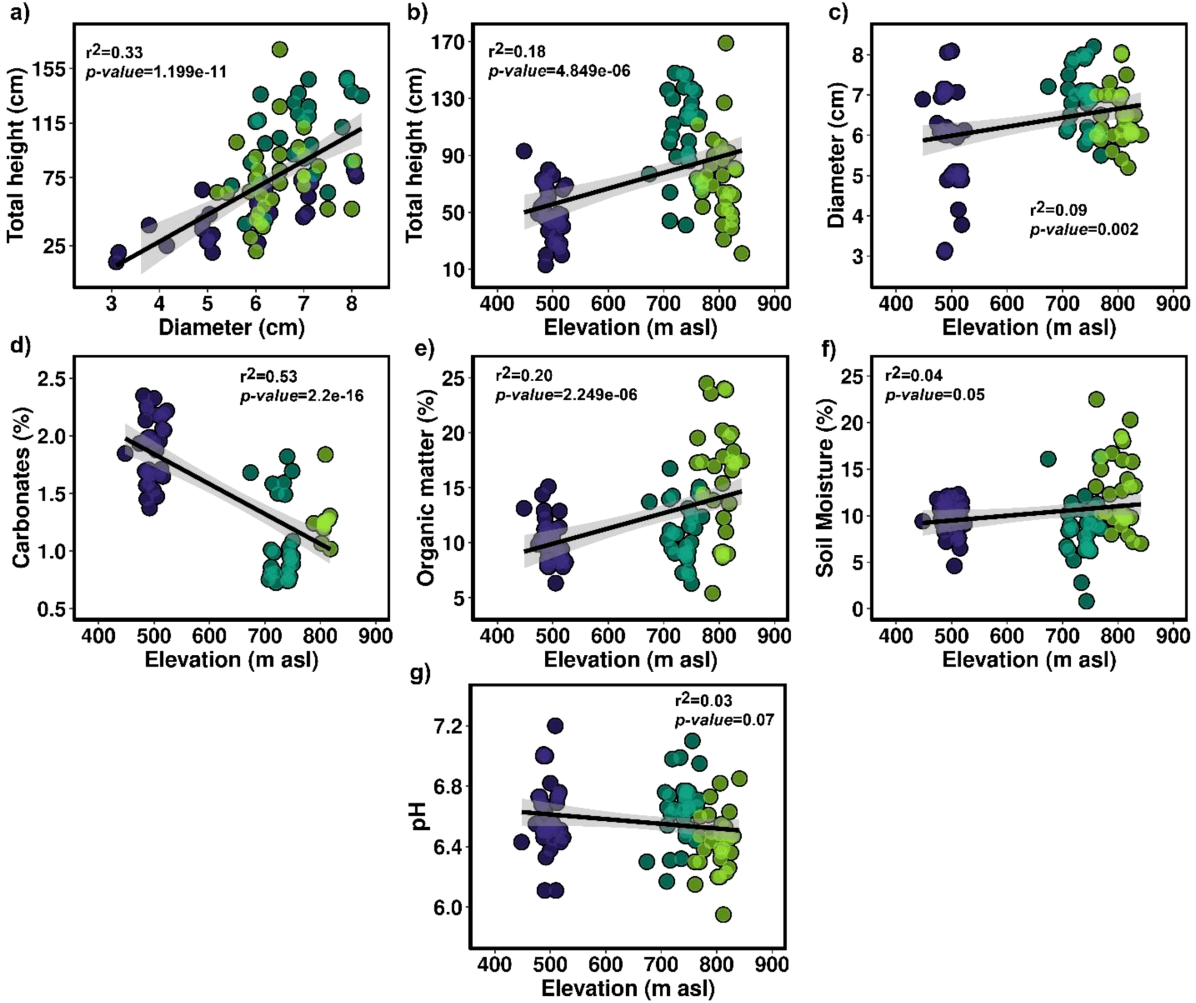
**(a)** Correlations between the diameter and total height of *H*. *acranthus*. **(b)** Correlations between elevation and the total height of *H*. *acranthus*. **(c)** Correlations between elevation and diameter. **(d)** Relationship between elevation and percentage of carbonates. **(e)** Correlation between altitude and organic matter. **(f)** Correlation between elevation and percentage of soil moisture. **(g)** Correlation between elevation and soil pH.

On the other hand, **Figure 7** illustrates a positive correlation between stem diameter and height (r² = 0.33, p-value = 1.199e-11). It is also reported that under the environmental conditions of the Lomas of Mangomarca, the maximum stem diameters and heights were 8 cm and 169 cm, respectively. Furthermore, it was observed that stems exceeding these reported maximum sizes tend to fall to the ground to continue their asexual reproduction processes. Additionally, correlations based on elevation indicate that more large cacti are found at higher altitudes (r² = 0.18, *p-value* = 4.849e-06), along with a higher concentration of organic matter (r² = 0.20, *p-value* = 2.249e-06) (**Figure 7**). In contrast, the percentage of carbonates decreases with increasing altitude (r² = 0.53, *p-value* = 2.2e-16) (**Figure 7**). These factors do not vary with elevation regarding stem diameter, pH, and soil moisture percentage.

## 4 Discussions

In this study, we report information on the spread, abundance, mortality, and structure of the *H. acranthus* species in a coastal lomas ecosystem. The results are concerning; despite the lomas of Mangomarca being considered a protected ecosystem, we counted approximately 14,741 dead colonies (**Figure 1**) and 1,801 living colonies over a total area of 3.79 ha along the altitudinal gradient (448 to 841 m asl) (**Figure 5**). Although the exact causes of death remain unknown, evidence suggests direct and indirect impacts of human activity, as the dead cacti were found at lower elevations and in areas near the city (**Figure 1**). Furthermore, human activities have intensified over the past 19 years, particularly due to the invasion. In 2020, they impacted 97.5 hectares out of 516.10 hectares (SERFOR, 2014), leading to a 46.9% reduction in vegetation cover (Bolivar and Velasquez, 2021). This has altered soil conditions, resulting in the death of many *H. acranthus* individuals, sensitive to changes in characteristics such as pH, EC, and OM (**Figure 4**), which have been drastically changed. Recent studies by Camel et al. (2024) also indicate that degraded soils affect the concentrations of Ca, Mg, K, and P, primarily due to soil disturbance from trampling by animals (packs of dogs), road opening, house construction, and other activities. Other studies also mention that cacti mortality is primarily due to prolonged periods of drought and extreme temperatures, such as frost (Orum et al., 2016), emphasizing the importance of nurse plants that protect against extreme temperatures and solar radiation. Similarly, our results indicate that at higher elevations, there is a greater concentration of organic matter and more prominent *H. acranthus* individuals; conversely, the lower areas exhibited a higher concentration of carbonates. These results align with previous studies, which indicate that as elevation increases in coastal lomas, the percentage of humidity rises (Dillon and Rundel, 1990), along with canopy cover, photosynthetic activity, and organic matter in the soil (Rolando et al., 2017). This facilitates the germination and growth of annual plants (Tovar et al., 2018), thereby boosting the nutrient cycle, particularly the organic matter cycle (Fabre et al., 2006). Furthermore, it is worth noting that *H. acranthus* individuals were found near rocks, as they play an essential role as nurse plants, capturing more humidity, reducing the temperature, preventing erosion, and providing micronutrients to other plants (Ángel et al., 2021).

Another important aspect is the adequate soil depth and presence of organic matter; according to our measurements, its average is 6.8 cm and is accompanied by small, non-rounded stone fragments mixed with gravel and coarse sand (Kalicki and Kalicki, 2020). This type of soil provides better access for establishing cacti, which have small roots. At a certain point, this also explains the scarcity of trees in the upper part of the lomas of Mangomarca. Similarly, another crucial variable negatively affecting the area is the pH; the results indicate that an increase in pH impacts the total size and the number of stems per colony. At lower elevations, there is more significant anthropic pressure due to illegal land invasions (Camel et al., 2024), and it is essential to note that packs of dogs have been observed inhabiting and moving through the hills (per. observation). During dry periods, substantial particulate matter rises, is carried by the wind, and settles on perennial plants’ biological soil crust (BSC) and the leaves and stems. These factors may alter the soil pH, leading to the progressive death of its biological organisms (**Figure 4**). Likewise, other studies (Gong et al., 2024; Sun et al., 2023) indicate that increased droughts and human activities negatively impact the characteristic pH of a soil type, altering the functionality of biodiversity (microorganisms, plants, animals, etc.) (Gong et al., 2024). Similarly, our results indicate that the soils of Coastal Lomas nearest to the road shift from acidic to alkaline pH, raising cacti mortality (**Figures 1c**, **4d**). The most concerning aspect is the death of BSC microorganisms, as they are linked to the decline in soil pH; this occurs because they release protons (H^+^) during photosynthesis (Jung et al., 2019), decompose organic matter (Chamizo et al., 2012), produce organic acids (such as oxalic and citric acid), and convert ammoniacal compounds into nitrates (Guida et al., 2023) all factors that contribute to acidification, especially in arid and semi-arid environments.

Despite the higher mortality rate recorded, structural studies enabled us to identify that in all colonies, there is a more significant number of stems between 20 and 40 cm, followed closely by those less than 20 cm (**Figure 5**). Additionally, in the highest areas, stems exceeding 1 meter were inventoried. At the same time, the diameter averaged a maximum of 8 cm. On the other hand, unlike adult trees that reach defined heights and experience a constant increase in diameter (Kessler et al., 2014), the *H. acranthus* species maintains a defined diameter even as the longitudinal size continues to grow until it reaches the length that allows it to lean parallel to the ground, thereby reproducing in an agamic manner (Arakaki et al., 2013). We consider this an essential strategy for the species to colonize new spaces, as they can root swiftly under suitable humidity conditions. Under experimental conditions, over 45 days, the stems rooted by 4.42 cm and grew an average of 4.80 cm for a year. This could be a significant strategy for expanding the production of *H. acranthus* individuals in nurseries. Moreover, it is essential to implement strategies for exchanging individuals between different coastal lomas to enhance genetic variability within the ecosystem (Arakaki et al., 2013). It should be noted that studies on *H. acranthus* plantations regard it as a potential bioremediation agent for arid soils, effectively reducing heavy metals such as zinc, antimony, and molybdenum from the soil, and may be beneficial in restoring coastal lomas ecosystems.

On the other hand, while polyploidy in the genus *Haageocereus* may help explain the phenotypic differences, life cycle, and development among species (Arakaki et al., 2007), further research is necessary to determine its reproductive mechanisms (Cota-Sánchez and Bomfim-Patrício, 2010). Recent findings suggest that in cacti, increasing seed size is closely associated with polyploid cytotypes (Cota-Sánchez and Bomfim-Patrício, 2010). Our reports on genome and seed size indicate that *H. acranthus* (2N = 4x = 44) contained 7.94 pg of DNA (**Figure 2**), while the seeds measured 1.51 mm in size and weighed 0.534 mg; these results were similar to those reported by Silva (2015). In contrast, the species *H. pseudomelanostele* (2N = 2x = 22) and *H. versicolor* (2N = 2x = 22) showed smaller seeds and lower amounts of DNA in pg (Arakaki et al., 2007; Silva, 2015). Future seed studies could support this hypothesis and provide additional information on recruitment, germination, and genetic diversity, among other factors. Finally, the results of the present study offer insight into the current state of conservation of the Mangomarca hills, highlighting the importance of propagating native species for the recovery of degraded areas. This way, the various species that depend on them can be protected.

## 5 Conclusions

This is the first study to document a species’ mortality levels and structure within the genus Haageocereus. In the Coastal Lomas of Mangomarca, 94 colonies were recorded across 4.41 ha, encompassing 1,801 stems. Of these, 37.89% of individuals had lengths between 20 and 40 cm, followed by plants smaller than 20 cm (24.82%). The largest stems of *H. acranthus* reached a diameter of 8 cm and a length of 169 cm. Across all hills, approximately 1,788 living cactus colonies and 14,741 dead colonies were counted. The mortality of cacti is likely attributed to anthropogenic impacts, as these modify the chemical and biological structure of the soil, changing its composition from acidic to basic, increasing electrical conductivity, and reducing both organic matter and nutrients. We also demonstrated that pH and altitude influence the phenotypic characteristics of *H. acranthus* stems. At higher elevations, the size of the cacti increased, along with the percentage of organic matter. At the same time, the concentration of carbonates decreased, likely due to the enhanced dynamics of annual plants during the southern winter. Additionally, *H. acranthus* has a genome size of 7.94 pg of DNA, with seeds averaging 1.51 mm in length and 0.53 mg in weight. Under nursery conditions, established stems grow approximately 4.8 cm per year, while the roots can develop up to 4.42 cm in just 45 days. Finally, mortality levels are expected to rise due to anthropogenic impacts’ severity, temperature increases and decreases in precipitation resulting from climate change. Therefore, careful monitoring is necessary, and conservation and restoration actions must be proposed for these coastal lomas that are endemic to South America.

## Data Availability

The raw data supporting the conclusions of this article will be made available by the authors, without undue reservation.
