# Supplementary material for: Mortality, structure, propagation, and microhabitat characterization of Haageocereus acranthus: a case study on coastal lomas
Source: Front Plant Sci. 2025 Jul 30;16:1577533. doi: 10.3389/fpls.2025.1577533 (PMC12344269; doi:10.3389/fpls.2025.1577533)
Supplement: Supplementary file 1 [file Table1.docx]

| Environmental variables | Transect-1 | Transect-2 | Transect-3 | Average |
| --- | --- | --- | --- | --- |
| pH | 6.6 | 6.65 | 6.43 | 6.36 |
| Organic material (%) | 10.18 | 10.99 | 15.93 | 12.37 |
| Slope (%) | 24.6 | 58.4 | 56.8 | 46.6 |
| Biological soil crust | 51.51 | 43.33 | 67.74 | 54.19 |
| Soil depth (cm) | 5.4 | 7.9 | 7.2 | 6.8 |
| Lichen presence (%) | 57.58 | 73.33 | 51.61 | 60.84 |
| Elevation m asl | 448-523 | 674-769 | 760-841 | 448-841 |
| Soil moisture (%) | 9.77 | 8.59 | 12.74 | 10.37 |

Table S1. Characterization of microhabitats according to transects.
